# FERONIA orchestrates P2K1-driven purinergic signaling in plant roots

**DOI:** 10.1080/15592324.2024.2370706

**Published:** 2024-06-21

**Authors:** Joel M. Sowders, Jeremy B. Jewell, Kiwamu Tanaka

**Affiliations:** a Department of Plant Pathology, Washington State University, Pullman, WA, USA; b Molecular Plant Sciences Program, Washington State University, Pullman, WA, USA; c Institute of Biological Chemistry, Washington State University, Pullman, WA, USA

**Keywords:** Extracellular ATP, purinergic signaling, purinoceptor, Ca^2+^ signaling, root growth, FERONIA, P2K1

## Abstract

Extracellular ATP (eATP) orchestrates vital processes in plants, akin to its role in animals. P2K1 is a crucial receptor mediating eATP effects. Immunoprecipitation tandem mass spectrometry data highlighted FERONIA’s significant interaction with P2K1, driving us to explore its role in eATP signaling. Here, we investigated putative P2K1-interactor, FERONIA, which is a versatile receptor kinase pivotal in growth and stress responses. We employed a FERONIA loss-of-function mutant, *fer-4*, to dissect its effects on eATP signaling. Interestingly, *fer-4* showed distinct calcium responses compared to wild type, while eATP-responsive genes were constitutively upregulated in *fer-4*. Additionally, *fer-4* displayed insensitivity to eATP-regulated root growth and reduced cell wall accumulation. Together, these results uncover a role for FERONIA in regulating eATP signaling. Overall, our study deepens our understanding of eATP signaling, revealing the intricate interplay between P2K1 and FERONIA impacting the interface between growth and defense.

## Introduction

In plant immune responses, receptors play crucial roles in detecting both external and internal cues. Pathogen-associated molecular pattern (PAMP) receptors are extensively studied because they recognize molecules associated with pathogens, triggering immune responses. In contrast, damage-associated molecular pattern (DAMP) receptors, which identify signals from damaged cells, have been characterized to a much lesser extent.^1^ Many DAMPs are direct products of the damaged self, arising from the pathogen infection process or physical damage. Core examples include sugar- or metabolite-derived molecules such as oligogalacturonides (OGs) and extracellular ATP (eATP): which are the product of cell wall breakdown and membrane rupture, respectively.^2–5^ Like PAMP-induced signaling, DAMPs employ some conserved early signaling events (i.e., Ca^2+^ influx, ROS production, canonical MAPK cascades). However, the receptor-interacting and downstream components in DAMP-receptor networks are largely unknown.^5,6^

Since the discovery of the eATP purinoceptor P2K1 a decade ago, numerous studies have investigated the protein-protein interactions of P2K1 to better understand the biochemical mechanisms of P2K1-mediated eATP signaling and DAMP signaling overall. In 2017, a significant milestone was reached with the identification of the first plant purinoceptor interacting protein. This research revealed that P2K1 directly phosphorylates the NADPH oxidase, RBOHD, in response to eATP, which regulates ROS production and subsequently influences stomatal immunity against the bacterial pathogen Pseudomonas syringae in guard cells.^7^

Our recent P2K1-interactomics dataset provided 121 putative P2K1-interacting proteins to explore.^8^ Among these proteins, FERONIA was identified as enriched with P2K1 in this dataset. FERONIA is a transmembrane kinase involved in various cellular processes which has recently been implicated in the maintenance of cell wall integrity.^9–11^ Thus, we were interested in exploring the involvement of FERONIA in the context of eATP signaling following our recent discovery of eATP’s role in regulating root growth by modifying cell wall morphology in the primary root tip.^12^ To this end, we evaluated eATP responses in the FERONIA mutant, *fer-4*, and found that loss of FERONIA perturbed eATP-responsive Ca^2+^ influx, gene expression, and cell wall modification.

## Methods

### Plant growth and materials

We employed wild-type Arabidopsis plants (specifically, the Columbia-0 ecotype, referred to as Col-0), *dorn1–3* mutant plants,^13^ *oxP2K1*_*t*_ (*35S:P2K1*_*t*_*-GFP*/*dorn1-3*),^12^ and *fer-4*.^14^ Each of the aforementioned plant germplasms also expressed the aequorin transgene (*35S:AEQ*).^15^ The seeds underwent surface sterilization before being placed on a solid growth medium. This medium consisted of ½ strength MS^16^ supplemented with vitamins (MSP09, Caisson, Smithfield, UT, USA), 1% (w/v) sucrose, 1% (w/v) daishin agar and was buffered using 0.05% (w/v) MES-KOH at a pH of 5.7. These seeds experienced a cold stratification treatment in darkness at 4°C for 3 days, followed by vertical growth in a growth chamber (CMP6010; Conviron, Winnipeg, Canada). The growth chamber maintained a temperature of 22°C, operated under a 12-hour photoperiod, and provided a light intensity of 100 µmol photons m^−2^ s^−1^.

### ATP treatment

For the root growth and RT-qPCR experiments, Arabidopsis plants were cultivated for 5 or 7 days, respectively. After the initial phase of vertical growth, seedlings were transferred to 6-well plates, accommodating 15–20 seedlings per well. These wells were filled with ½ MS medium without agar (referred to as “liquid media”) for RT-qPCR assays, or with agar plates containing or lacking ATP for root growth assessments. An ATP stock solution (100 mM) was prepared by dissolving ATP (Sigma-Aldrich. St. Louis, MO, USA) in 50 mM MES buffer. The pH of this solution was adjusted to 5.7 using KOH, and after filter sterilization, it was stored in aliquots at − 20°C for indefinite use. Unless specifically mentioned, exogenous ATP (eATP) was administered at a final concentration of 500 μM. This concentration has recently been established as the optimal working concentration to maximize measurable responses to extracellular ATP.^12,17–19^

### Co-immunoprecipitation-coupled mass spectrometry (Co-IP/MS)

Co-IP/MS analysis involved grinding seedlings with sand in liquid nitrogen-cooled mortar, followed by suspension in ice-cold buffer H. This buffer consisted of 50 mM HEPES (pH 7.5), 250 mM sucrose, 15 mM EDTA, and 5% (w/v) glycerol, supplemented with 1 mM DTT, 1X HALT protease/phosphatase inhibitor cocktail (Thermo Fisher Scientific, Waltham, MA, USA), 50 µM MG132, and 10 nM calyculin.^20^ After centrifugation, supernatants were filtered and combined with microsomal pellets. The microsomal fraction was resuspended in 0.75 mL buffer H containing 0.5% IGEPAL CA-630 (from a freshly-prepared 10% stock solution), and subsequently diluted to a final volume of 1.5 mL using buffer H without IGEPAL. GFP-Trap beads were incubated with extracts, washed, and eluted for SDS-PAGE. Eluates were sent for mass spectrometry analysis. Scaffold software version 4^21^ analysis was used to compare peptide enrichment differences between P2K1-GFP and GFP-LTI6B samples. For more information, see the P2K1 interactome dataset released by Sowders et al.^8^

### Root growth measurements

The change in root length following the transfer of 5 d old Arabidopsis plants to mock (MS) or 500 µM ATP containing plates for 24 h was quantified with ImageJ (version 1.53q) from photographs taken by an EPSON V600 scanner (800 dpi).

### Calcium measurement

The described germplasms (expressing aequorin) were germinated on ½ MS media with agar. Three days after germination, the seedlings were moved to a 96-well plate. Each well contained 50 μL of coelenterazine buffer composed of 2 mM 2-ethane sulfonic acid (MES) at pH 5.7, 10 mM CaCl2, and 10 μM coelenterazine. The plate was then kept in darkness overnight. The next day, the plates were placed in the GloMax® Navigator plate reader (Promega, Madison, WI, USA). Subsequently, the seedlings in the wells were treated with 100 μM ATP (buffered with MES to pH 5.7) using the onboard injector. Luminescence was recorded for 80 seconds after treatment, and the calcium influx was quantified using a previously described method.^22^

### RT-qPCR

Seven-day-old seedlings were moved to ½ MS liquid medium and equilibrated overnight. The next day, plants were treated with liquid medium alone or with ATP (added to achieve 500 μM final concentration) for 30 min. Plants were harvested, frozen in liquid N2, and stored at − 80°C. Tissue was homogenized with a Mini-Beadbeater™ (BioSpec Products, Bartlesville, OK, USA). RNA isolation used the Quick-RNA kit (Zymo Research, Orange, CA, USA) and RNA concentration was assessed with Eppendorf μCuvvete® G1.0 on a BioPhotometer® D30 (Eppendorf, Hamburg, Germany). cDNA synthesis began with 1 μg RNA and iScript kit (Bio-Rad Laboratories, Hercules, CA, USA), followed by a 10-fold dilution of cDNA. SYBR Green reaction mix (SsoAdvanced; Bio-Rad) was used with 2 μL of cDNA for a 20 μL reaction in a CFX96 thermocycler (Bio-Rad). Target gene expression was normalized to PP2A.^23,24^ Statistical analysis employed two-way ANOVA with Tukey post hoc test in GraphPad Prism V.8, as indicated for individual experiments.

### Staining and microscopy

Col-0 and *fer-4* seedlings were germinated (described above) and grown for 5 days. The seedlings were then transferred to mock (MS) or 500 µM ATP-containing plates for 3 days. After the treatment, the seedlings were incubated in an aqueous solution of 1% calcofluor white (CFW) for 5 minutes,^25,26^ washed thrice with PBS, and imaged with a Leica DMI6000 microscope (Leica, Wetzlar, Germany).

## Results

**Figure 1.**
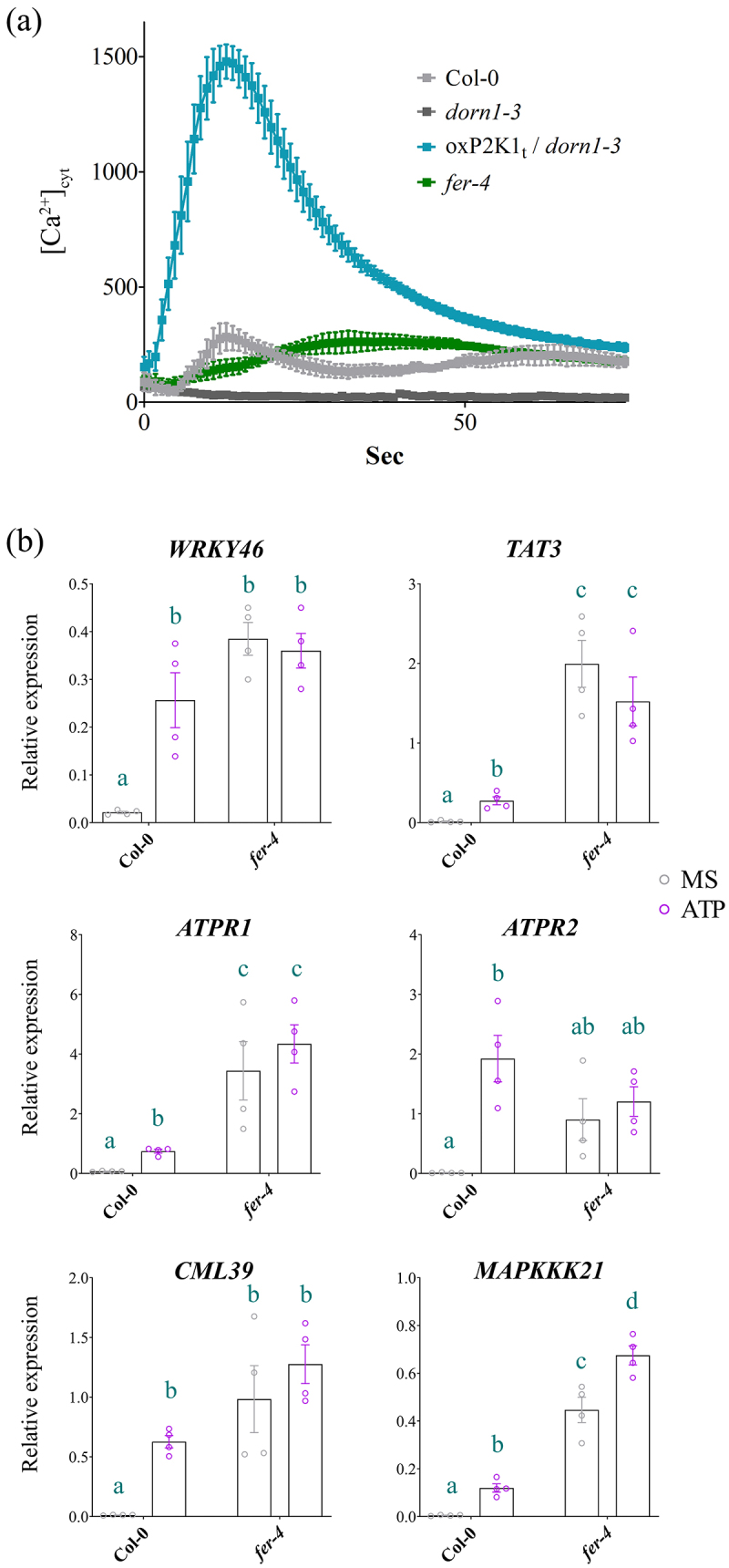
eATP-induced calcium influx and gene expression hallmarks are perturbed in *fer-4* (a) Calcium measurements were conducted using aequorin luminescence in Arabidopsis genotypes expressing *35S:AEQ*. The measurements were taken for 80 sec after treatment with ATP (100 μM). (b) eATP-responsive gene expression was measured 30 m after mock or 500 µM ATP treatment in 7 d old Col-0 or *fer-4* plants. All gene expression values were normalized to *PP2A*. n = 4 reps (15 seedlings/rep). Statistical analysis was conducted using two-way ANOVA with Tukey post hoc test. Samples without overlapping letters indicate significant differences (p < 0.05). Error bars = SEM. All experiments were repeated thrice with consistent results.

The putative P2K1-interacting receptor kinase, FERONIA, was identified in our Co-IP/MS dataset (Table 1^8^; as significantly enriched with P2K1-GFP compared to negative control, GFP-LTI6B (*p* = 0.0041; Fisher’s exact test). To assess the impact of FERONIA on P2K1-mediated eATP signaling we obtained the loss of FERONIA null mutant, *fer-4*, from the Arabidopsis Biological Resource Center (ABRC), which had originally been noted for root hair and auxin signaling defects.^14^ First, we evaluated calcium influx in response to eATP in the *fer-4* mutant plants. We found that *fer-4* plants exhibited an atypical calcium response to eATP compared to wild type, Col-0, plants (Figure 1(a)). Meanwhile, the *P2K1* overexpression (*oxP2K1t*) and loss of P2K1 mutant (*dorn1–3*) plants had exaggerated and absent eATP responses, respectively, as expected (Figure 1(a)).

**Table 1.** FERONIA is enriched with P2K1-GFP.

		P2K1-GFP			GFP-LTI6B	
			+ATP			
Locus	Name	Mock	3 m	20 m	Mock	
AT3G51550	FERONIA	3.585	3.585	2.807	1.585	

Co-immunoprecipitation tandem mass spectrometry (CoIP-MS) of P2K1-GFP or negative control protein, GFP-LTI6B from the published dataset.^8^ FERONIA was significantly enriched with P2K1-GFP samples compared to GFP-LTI6B (*p* = 0.0041; Fisher’s exact test). Protein abundance is reported as log2 peptides identified. See Sowders et al.^8^ for additional descriptions.

Next, we looked at eATP-responsive marker gene expression^17,18^ in the *fer-4* mutants. Notably, we found that 5 out of 6 (*WRKY46*, *TAT3*, *ATPR1*, *CML39*, and *MAPKKK21*, but not *APTR2*) eATP marker genes tested were constitutively upregulated in *fer-4* plants compared to Col-0 (Figure 1(b)). Although 83% of the eATP marker genes were significantly upregulated in *fer-4* compared to Col-0 under basal (mock) conditions, only one of the genes, *MAPKKK21*, was differentially expressed following the application of ATP in *fer-4* (Figure 1(b)). A possible indication that the expression level of eATP-responsive genes is at or near saturation in the *fer-4* mutant.

**Figure 2.**
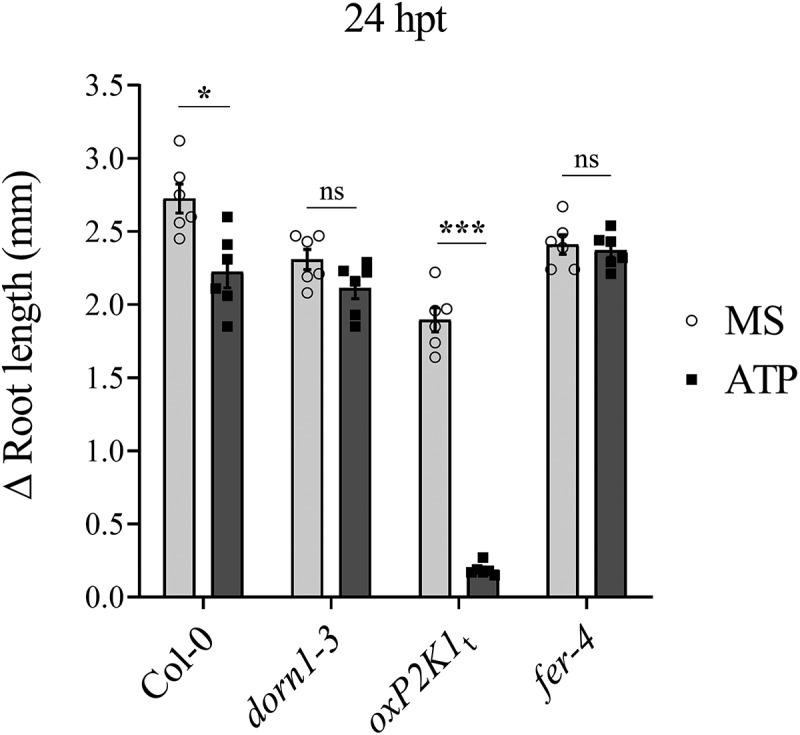
*fer-4* is insensitive to eATP-regulated root growth Five days after germination on ½ Murashige and Skoog (MS) media, seedlings of given genotypes were transferred to media containing ½ MS or ½ MS with 500 μM ATP. The change in root growth was measured 24 hours post-transfer (hpt).Genotypes: FERONIA null mutant, *fer-4*; P2K1 null mutant, *dorn1–3*; C-terminal truncated P2K1 overexpression, *oxP2K1*_*t*_^12^, and Col-0 wild-type Arabidopsis. Three to four biological replicates; n = 10–15 seedlings per replicate, error bars = SE. ANOVA with Tukey multiple comparison tests was used to determine statistical significance in Graph Pad Prism (8.0.2). Asterisks distinguish between significant thresholds (*p < 0.05; ***p < 0.001). The experiments were repeated at least three times with similar results.

**Figure 3.**
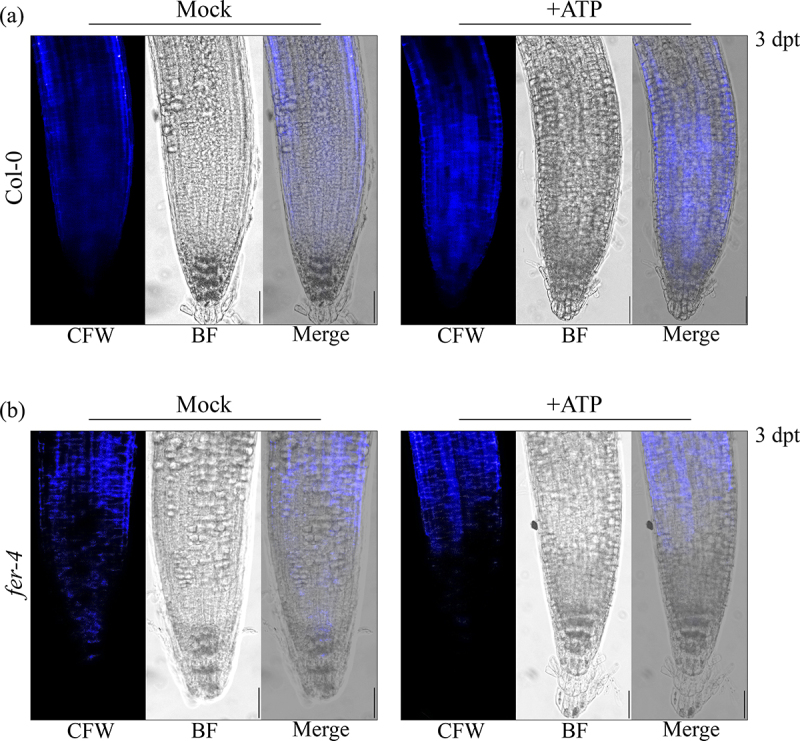
*fer-4* is insensitive to eATP-regulated cell wall deposition Cell wall morphology was evaluated in (a) Col-0 and (b) fer-4 by calcofluor white (CFW) staining 3 days post-treatment (dpt) with mock (MS) or 500 μM ATP. Scale bar = 150 µM. The experiments were repeated thrice with similar results.

To further investigate the impact of FERONIA on P2K1-mediated eATP signaling, we investigated eATP-regulated root growth and cell wall integrity in *fer-4*. Loss of FERONIA in *fer-4* mutant plants resulting in insensitivity to eATP-regulated root growth relative to Col-0 which is reminiscent of the eATP-insensitive P2K1 mutant, *dorn1–3* plants (Figure 2, S1). Considering wild type and *oxP2K1* displayed an increase in cell wall accumulation following eATP treatment,^12^ we postulated that *fer-4* plants may be insensitive to eATP-regulated root growth because they fail to accumulate the cell wall material that leads to the increased cell wall rigidity that suppresses root elongation.^12^ To test this hypothesis, we transferred *fer-4* plants to mock or ATP-containing media for 3 days and stained them with the cell wall stain, calcofluor white. Consistent with our hypothesis, *fer-4* plants displayed less cell wall accumulation 3 d after eATP treatment compared to Col-0 (Figure 3).

## Discussion

Over the past 20 years, extracellular ATP has been recognized as a critical signaling molecule in both animal and plant physiology. Its role as a purinergic signal in plant growth and defense is now well-established within the plant biology community. However, despite the identification of a few interactors of eATP receptor P2K1,^7,27–29^ the lack of a comprehensive interactome for P2K1 and the limited methods for monitoring eATP responses have hindered a complete understanding of purinergic signaling mechanisms. While our recent interactomics study identified several potential interacting partners of P2K1, including CPK28,^8^ further exploration of these interactions and their functional significance is warranted.

Past studies have shown that eATP-induced expression of certain genes intersects with the signaling hormone, jasmonic acid (JA).^17,19^ Our recent study suggested that eATP sensing may feed into the abscisic acid (ABA) signaling pathway to regulate primary root growth.^12^ In the same study, we demonstrated that cell wall accumulation in the primary root tip is a possible mechanism for regulating root growth downstream of eATP perception. FERONIA is a central receptor kinase with cellular activities intersecting ABA,^14,30^ JA,^31^ and cell wall integrity^9–11,32^ among other processes determining the balance between plant growth and defense.^33–35^ Thus, FERONIA immediately became an intriguing interacting candidate protein to evaluate in the context of purinergic signaling. Here, we have provided a proof-of-concept evaluation of the impact of the putative P2K1-interacting component, FERONIA, on eATP signaling physiologies. In summary, we found that loss of FERONIA in *fer-4* mutant plants had disturbed eATP-induced Ca^2+^ mobilization and gene expression (Figure 1) and that *fer-4* plants were insensitive to eATP-regulated root growth (Figure 2) and cell wall deposition (Figure 3).

Although eATP-responsive genes showed higher expression in *fer-4* plants, they mostly did not respond to eATP treatment (Figure 1). Similarly, when exposed to eATP, *fer-4* plants did not exhibit cell wall deposition in the primary root tip or a reduction in root growth rate (Figures 2, 3). Given *fer-4* was insensitive to eATP-responsive physiology, we suppose that FERONIA might be a positive regulator of eATP-responsive cell wall deposition and a negative regulator of eATP-responsive gene expression. According to this speculative model, FERONIA could be a multifaceted regulator of ATP signaling, potentially through direct interaction with P2K1.

The constitutive upregulation of eATP-responsive genes in *fer-4* plants might be explained by the absence of FERONIA activity. For instance, FERONIA might promote eATP-responsive physiological responses while also functioning in a negative regulatory loop to “turn off” eATP-responsive gene expression after receiving the signal and regulating cell wall deposition. Thus, although eATP-responsive gene expression may already be saturated in *fer-4* plants, the characteristic eATP-triggered physiological responses fail to occur without FERONIA activity. Alternatively, given that JA- and eATP-responsive transcriptional changes largely overlap and depend on the MYC2/MYC3/MYC4 transcription factors,^13,17,19^ and that loss of FERONIA function has been reported to stabilize the MYC2 transcription factor and constitutively induce JA-responsive genes,^31^ the gene expression differences noted here may be independent of eATP signaling. While the exact role of FERONIA in regulating eATP-responsive gene expression is not entirely clear, FERONIA does appear essential for early and downstream physiological responses to eATP, such as calcium mobilization, and cell wall deposition and root growth regulation, respectively. Further investigating the interplay between eATP, hormone-mediated responses, and FERONIA could provide valuable insights into the complex cell-surface receptor networks regulating plant growth and defense.

## Supplementary Material

Figure S1.tif

## Data Availability

The data that support the findings of this study are available as published in JMS’ doctoral dissertation,^36^ and from the corresponding author [kiwamu.tanaka@wsu.edu] upon reasonable request. The plant germplasms generated in this study will be made available upon request and shared in compliance with the relevant governing bodies.
