# Supplementary figures and images for: FERONIA orchestrates P2K1-driven purinergic signaling in plant roots
Source: Plant Signal Behav. 2024 Jun 21;19(1):2370706. doi: 10.1080/15592324.2024.2370706 (PMC11195479; doi:10.1080/15592324.2024.2370706)

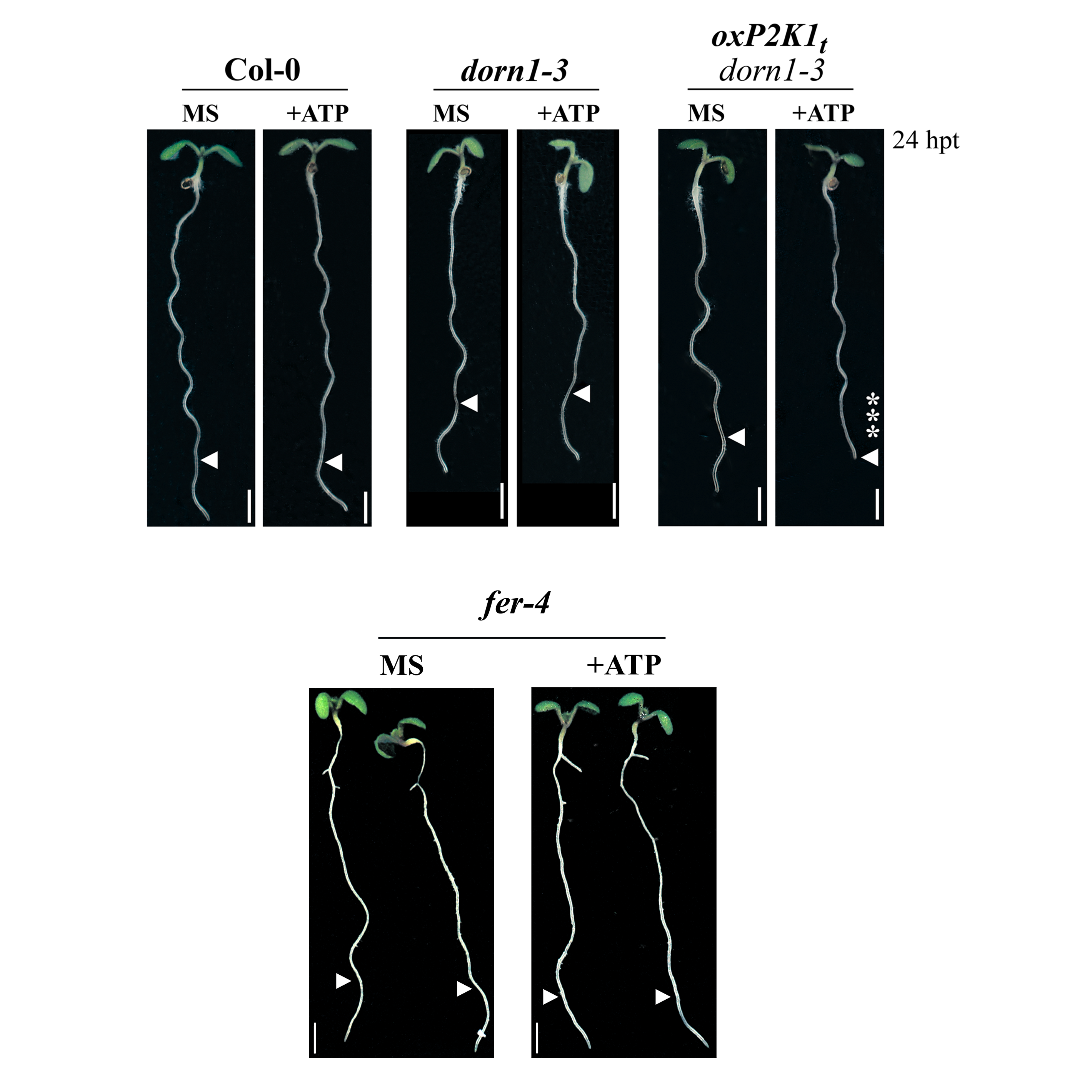

Supplement: Figure S1.tif [file KPSB_A_2370706_SM8183.tif]
